# The Institutional Library

**Published:** 1920-04-03

**Authors:** 


### THE INSTITUTIONAL LIBRARY.

**A Text-Book of Gynæcological Surgery.** By Comyns Berkeley, M.D., F.R.C.P.; and Victor Bonney, M.D., F.R.C.S. (Cassell and Co. Second Edition. 1920. Pp. 829. Price 42s. net.)

Since its first appearance nine years ago this text-book has been recognised as the most lucid and the most comprehensive survey of modern British operative gynæcology. It was reprinted twice, and the authors state that a second edition would have been published in 1914 but for the war. As now reissued it justifies in full the appreciative review of the first edition which appeared in these columns at that time. As before, care, thoughtful reasoning, clear thinking, and apt expression are traceable from beginning to end. The new sections are fully on the same high levels as the old ones; thus there now appears a chapter on abdomino-perineal excision of the rectum, which is quite the best description of that complicated operation so far published. There is a chapter on saline and blood infusion in which the account of blood transfusion appears to have been added somewhat as an after-thought, and no pronouncement is made as to whether this method of treating shock is more or less effective than the older saline methods; the amount of blood to be transfused is not given, and gum saline solution is not mentioned. In a later chapter on the treatment of shock there is a discussion of the relative values of these remedies, and of other means of combating shock; these two chapters ought to be mutually cross-referenced. We are pleased to see that the authors approve of spinal anæsthesia, combined with very light general anæsthesia, for severe pelvic operations, such as Wertheim's hysterectomy; this is a valuable method which British surgeons have been rather too conservative in adopting. An outstanding text-book, and a credit alike to authors, publishers, and British surgery.

**Chemistry for Public Health Students.** By E. Gabriel Jones. (London: Methuen and Co., Ltd. Price 6s. net.)

In a thoroughly practical little book, within the small compass of 244 pages, Mr. E. Gabriel Jones has managed to condense all that is necessary for students working up the practical chemistry for the D.P.H. He has also done rather more than this, for he has dealt with many matters—especially in the food chapters, which, though hardly likely to be asked at the examination, will nevertheless be found extremely useful to medical officers of health. The paper is of good quality, the print large and very clear—qualities which everyone will appreciate in these days of poor paper and bad print. Students will also be grateful for many useful elementary explanations dealing with chemical and mathematical formulæ, which are often omitted in more advanced works; for it is often the painful experience of teachers of chemistry and physics that mathematics are not the strong point of the ordinary medical student, or that even did he possess a little extra skill in these subjects on his entry into hospital, this has disappeared by the time he comes to study for the D.P.H.

**A Medical Handbook for the Use of Practitioners and Students.** By R. S. Aitchison, M.D., F.R.C.P.E. With numerous illustrations. Published in London by Charles Griffen & Co., Ltd., Exeter Street, Strand, W.C. 2. Price 10s. 6d. net.

The present edition of this very useful little book contains much that is both new and valuable to the practitioner, and the author has obviously taken considerable care to bring the work as far as possible up to date. Complicated laboratory tests are mentioned, and the moments when they become essential carefully indicated, but it is satisfactory in a book of this type that first and foremost is the question of *clinical diagnosis* considered, and we therefore heartily recommend Dr. Aitchison's fifth edition to the busy practitioner and the student alike.

**On Facial Neuralgia and its Treatment.** By J. Hutchinson, F.R.C.S. (London: John Bale, Sons and Danielsson. 1919. Pp. 216. Price 15s. net.)

This monograph represents the revised and expanded essay on the subject for which Mr. Hutchinson was awarded the Jacksonian Prize in 1915. The whole of the important subject named is taken under review; but far the greater part of the book is devoted to that intractable, maddening form of pain which has been christened "epileptiform neuralgia," and to its operative treatment. The whole book is most valuable and timely, and should be a tower of strength to general practitioners and surgeons alike. The only fault to be found with it, considering the inexpensive format, is the exorbitant price at which it is put on the market, which will, it is to be feared, militate against a wide circulation.

**Handbook of Anæsthetics.** By J. Stuart Ross, M.B. (E. & S. Livingstone. 1919. Pp. 214. Price 7s. 6d. net.)

To summarise an important specialty of medicine, itself comprising an art as well as a great deal of science, within the covers of so small a book as this is no mean feat. Difficult as it is, Dr. Stuart Ross has accomplished it extremely well. There is much in the subject which he has had to leave out, and many important points on which he has had to be extremely brief. But he has exercised a discretion which has mostly been well advised, and his task has been well done. Those who do not want an encyclopædic treatment of the subject, but are satisfied with plain and authoritative teaching on practical matters, briefly condensed, will find here as good a small textbook on anæsthetics as we know of at present. It is noticeable that the author has gone to a surgeon for chapters on local and spinal anæsthesia. Though the technique and practice of local anæsthesia are usually, and rightly, practised by surgeons and not by their anæsthetists, yet it does seem that spinal anæsthesia should remain the province of the latter, and not of the former.

**Engines of the Human Body.** By Professor Arthur Keith, M.D., F.R.S. (Williams and Norgate. 1920. Pp. 283. Price 12s. 6d. net.)

Professor Keith can and does make of anatomy what it should be, but very seldom is made, a science of absorbing interest both to medical men and the general public. He has a positive genius for the popular exposition of highly technical subjects, both in the lecture-room and in print. This present volume is founded upon a series of lectures given during the war at the Royal Institution, and is really a treatise on human physiology made understandable of the many, and made fascinating as well. Professor Keith admits having had the great example of Faraday before him when he accepted the invitation to become a Royal Institution lecturer; and it is only just to admit that he reaches the Faraday ideal more nearly than any scientist of the day that we know of. This book can be heartily recommended to all and

**The Institutional Library**—(*continued*).

sundry; we believe that even veteran medical practitioners will find instruction in it, and we are quite sure that even children will find it interesting and intelligible.

**Massage and the Original Swedish Movements.** Their Application to Various Diseases of the Body. By Kurre W. Ostrom. Eighth edition, revised and enlarged, with 125 illustrations. (London: H. K. Lewis and Co. 1919. Price 5s.)

The writings of K. W. Ostrom are already well known to all students of massage and Swedish remedial exercises, and the eighth edition of his work, revised and enlarged, together with some modification of previous statements, sixteen entirely new pictures and others re-drawn, will prove as interesting and instructive as his previous publications. The present volume is a clearly expressed treatise on the art as practised by Swedish manipulators. It is an explanation of the performance of the various movements in their application to all parts of the body, and also of the diseases for which they are indicated or contra-indicated. It is not a text-book for the qualifying student, but is of great assistance and of practical value in the treatment of disease by a therapeutic system of remedial exercises.

**Obstetric and Gynæcological Nursing.** By Edward Davis. (Philadelphia and London: W. B. Saunders. $2 net.)

This book is not intended for midwives but for monthly and gynæcological nurses working under a doctor, that they may be able to do their part intelligently and to understand the principles on which they are working. The advice to nurses concerning the hygiene of pregnancy will be found very useful, though the amount of fresh air suggested seems rather limited to English ideas. It is only in cold or windy weather that a room can be ventilated sufficiently by putting windows on the block. Sea-bathing also seems a slightly rash proceeding for a pregnant woman. It should have been made clear that if the nurse has any reason to suspect that the uterus is "tipped backwards," as the author expresses it, she should report at once to the doctor. The condition may, of course, relieve itself as the uterus rises out of the pelvis, but the nurse should not take the responsibility for such a case. The section on the toxæmia of pregnancy is very practical, and that dealing with labour will be found useful by monthly nurses, though a somewhat misleading statement is made as to the end of the first stage. This is not necessarily "marked by the rupture of the membranes," which often takes place either earlier or later. The directions in the emergencies which may arise in labour cases, when the doctor has not arrived, should be a help to the nurse. The sections on surgical operations and dressings give very full directions, and the nurse who studies them ought to be well up in all aseptic methods. The illustrations are clear and good.

**A Challenge by Lt.-Col. Maitland Hardyman, D.S.O., M.C.,** with a Foreword by Norman Hugh Ronanes. (London: George Allen and Unwin, Ltd.)

These verses were written by a young man who was killed in his twenty-fourth year, after being rapidly promoted. He enlisted in August 1914, and after holding various staff appointments was killed in action in August 1918. From the Foreword we gather that Hardyman had a strong personality, which impressed itself upon all who came in contact with him, and indeed is evident from the fact that while condemning in principle the action of conscientious objectors, he was elected on the Council of the Union of Democratic Control, while he retained the confidence of his military superiors. He fought as many fought—without illusion, and his attitude is summarised in the vigorous line:

"The Jingo's claptrap is his own rebuke."

It is this vigour which lends such distinction as his verse possesses, and in such poems as "From a Hospital in Suez" we get the now familiar expression of the confusion in the mind of an average soldier who is unable to see the wood for the trees. The verses will be appreciated as a memorial by those who knew him, but in themselves they do not display any particular poetic gift.

### TWO BOOKS FOR HOLIDAY READING.

**Diversions in Sicily.** By H. Festing Jones. (London: A. C. Fifield. Price 6s. net.)

**Castellinaria.** By H. Festing Jones. (London: A. C. Fifield. Price 6s. net.)

Now that Mr. Festing Jones has taken his seat beside Boswell with the publication of his masterly life of Samuel Butler (which was reviewed in The Hospital on November 1, 1919), it is well that the public should be reminded of his earlier writings by the present reissue of "Diversions in Sicily" and "Castellinaria," two of the most pleasant holiday books of recent years. A vivid interest was given to "Diversions in Sicily" by the presence in London, about the time when it first appeared, of Signor Grasso and the Sicilian players; for "Diversions" is full of their doings, and when Mr. Jones wrote about them, as their familiar friend, he possibly had no idea that we should eventually make their acquaintance in Shaftesbury Avenue, since London is usually the last place which men of European reputation are invited to visit. It is probably safe to say that no books about Sicily have been written like these. When Mr. Festing Jones visits a ruin you hear more about his driver and his picnic than the place which was the excuse for his diversion. It is the Sicilian character which most attracts him. The climate, history, and beauty of the island are but the background on which the Sicilians disport themselves. He speaks of Sicily as his second country, and he tells you of the interests of the peasants, not of the sights which the tourist goes to see. It is like reading a series of letters from a friend who is enjoying himself and takes his pleasure with the people of the place. Their child-like quality, frankness, and open manners appeal to his sense of humour, and he becomes one of them for a season. His humour plays over his pages in passages like that wherein he confesses that there is much ado about little in the eating of an artichoke. "Artichokes," he proceeds, "as a means of passing the time on Sunday reminded me of the Litany: pulling off each leaf was like listening to each short clause, and eating the unimportant little bit at the end was like intoning the little response." The diversions, then, which are continued in "Castellinaria," are the diversions of the Sicilians themselves. In the second book we read of the marionettes and their theatre, and of a hundred-and-one characters and scenes in the medley of Sicilian daily life. It is the story of a succession of holidays taken without ulterior motives, and so written, we imagine, as to delight the people whose fun and good-fellowship are recorded. The volumes are as hard to describe as a book by George Borrow, or Mr. W. H. Davies' autobiography; for they are about men, not monuments; about life, not about things; and give a picture of Sicilian manners which in freshness and simplicity tell one more about the island than a hundred travel books, which would not have been written at all if the writers had not thought beneath their notice everything omitted from the guide-book. This omission is here repaired, and the process is diverting.

